# Supplementary material for: Revealing new pathways for the reaction of Criegee intermediate CH2OO with SO2
Source: Commun Chem. 2024 Jul 13;7:157. doi: 10.1038/s42004-024-01237-9 (PMC11246420; doi:10.1038/s42004-024-01237-9)
Supplement: Supplementary file 2 — Description of Additional Supplementary Files [file 42004_2024_1237_MOESM2_ESM.pdf]

Description of Additional Supplementary Files

File name: Supplementary Data 1

Description: Optimized Cartesian coordinates (in angstrom) and energies (in atomic units) obtained on the PES
